# Developing and establishing the psychometric properties of the Strathclyde Citizenship Measure: A new measure for health and social care practice and research

**DOI:** 10.1111/hsc.13789

**Published:** 2022-03-28

**Authors:** Nicola Cogan, Gillian MacIntyre, Ailsa Stewart, Hilary Harrison‐Millan, Karen Black, Neil Quinn, Michael Rowe, Maria O’Connell

**Affiliations:** ^1^ 3527 Psychological Science and Health University of Strathclyde Glasgow UK; ^2^ 63897 Turning Point Scotland Glasgow UK; ^3^ Yale University New Haven Connecticut USA; ^4^ Social Work and Social Policy University of Strathclyde Glasgow UK

**Keywords:** citizenship, health and social care, life disruptions, mental health, psychometric validation, recovery; measure development

## Abstract

There has been increasing interest and research attention towards citizenship‐based practices and care within health and social care settings. A framework for implementing citizenship‐based interventions has helped support the participation in society of persons who have experienced major life disruptions. Yet, having ways to measure the impact of citizenship ‘in action’ within specific socio‐cultural contexts has proved challenging. We report on the development of the Strathclyde Citizenship Measure (SCM) which seeks to establish a psychometrically sound measure of citizenship that is relevant to the Scottish context. We outline the three phases of developing the SCM: (1) item generation, (2) item reduction and piloting, and (3) measure validation. Having generated items for the SCM using concept mapping techniques, we piloted it with 407 participants who completed an online survey of a 60‐item version of the SCM. The aims were to assess the validity of the items and reduce the number of items using principal components analysis for the final measure. This resulted in a 39 item SCM. We then sought to establish the psychometric properties of this shorter version of the SCM through testing its reliability, convergent, concurrent and discriminant validity. The 39 item SCM was administered online to 280 Scottish residents along with additional measures including the Warwick‐Edinburgh Mental Well‐being Scale (WEMWBS), the Depression, Anxiety and Stress Scale (DASS21), the Sense of Belonging Instrument (SOBI‐A); the Big Five Personality Inventory (Shortened Version; BFI‐10) and the Personal Social Capital Scale (PSCS‐16). The factor structure and dimensionality of the SCM was examined using exploratory factor analysis and it was found to be reliable and valid. This paper explores the potential for the application of the SCM across health and social care settings and identifies future work to develop citizenship tools to facilitate dialogues about citizenship across health and social care practice settings.

**What is known about this topic?**

- Citizenship is a much‐theorised, multi‐dimensional concept yet its application in health and social care practice is relatively novel.
- Citizenship‐focused practice has much to offer individuals and groups who have been marginalised and excluded from communities and mainstream society by shifting the focus from individual deficits to broader structural and social aspects of citizenship.
- The extent to which the benefits experienced by individuals engaged in citizenship‐based intervention programmes is not well understood and has been difficult to capture using existing outcome measures which are often focused on symptoms and illness‐based constructs that do not adequately capture the aspects of citizenship that people identify as important to them.

**What this paper adds?**

- This paper adds greater understanding of the aspects of citizenship that are important to individuals with major life disruptions via the development of a new 39 item Strathclyde Citizenship Measure (SCM) which has been shown to be a reliable and valid measure.
- The SCM can be used to evaluate the outcome of intervention programmes that aim to increase a sense of citizenship across practice and community settings.
- The SCM can be used as a reflective tool to support practitioners to gain insight into their own experiences of citizenship and to develop constructive dialogues about citizenship with people who use services thus promoting better partnership working and relationship‐based practice.

## 1 INTRODUCTION

Citizenship is emerging as a promising and significant framework internationally that promotes a response to mental illness that shifts the focus from traditional medical models of mental illness and psychiatric concepts in clinical settings into community programmes with a renewed focus on social inclusion and the full participation of individuals with lived experiences (Reis et al., 2022; Rowe & Ponce, 2020). Citizenship is often associated with the concept of recovery (Davidson et al., 2021; Nesse, Aamodt, et al., 2021; Rowe & Davidson, 2016) and can be understood as a useful framework for the integration and involvement of marginalised, ‘seldom heard’ or ‘easy to ignore’ individuals in their communities thus enhancing individual recovery (Lightbody, 2017; MacIntyre et al., 2021; Pelletier et al., 2015; Rowe, 2015). This is particularly important given that these groups are often the most marginalised and excluded, facing multiple sources of disadvantage and inequality (Cogan et al., 2021; Hamer et al., 2014; Lightbody, 2017). The citizenship framework can provide the basis for understanding the key components necessary to achieve equal status as a member of one's community (Ponce et al., 2012; Rowe, 2015). Experience of mental ill health, substance misuse, chronic physical health problems, homelessness, drug and alcohol misuse and/or forensic involvement often result in significant life disruptions that can lead to socioeconomic disadvantage and other forms of exclusion (Cogan et al., 2021; Davidson et al., 2021; McNiel et al., 2005). Such disruptions can negatively impact a person's engagement in society and have consequences such as reduced access to health care, employment, civic rights and responsibilities; resulting in the loss of a person's sense of citizenship.

Research on the concept of citizenship has been increasing (Davidson, 2016; Quinn et al., 2020), however, the application of citizenship across health and social care settings is relatively novel (MacIntyre et al., 2021; Morgan et al., 2020). While diverse conceptualisations of citizenship exist (Beresford & Croft, 2016; Dean, 2004; Isin & Nielsen, 2008; Lister, 1998; Marshall, 1964; Yuval‐Davis, 2007), citizenship theory and practice has traditionally focused on securing the rights of the individual within society. In more recent work, ideas around collective citizenship have been developed that integrate the community and structural dimensions of citizenship, including the role of advocacy and challenging power structures that disempower and discriminate against people within marginalised groups (Beresford, 2013; Beresford & Croft, 2016; Cogan et al., 2021; MacIntyre et al., 2021; Quinn et al., 2020; Stevenson et al., 2015).

Recent work within the United States (U.S) has aimed to bring together ideas around individual and collective citizenship through the framework of the 5Rs of citizenship: the rights, responsibilities, roles, resources, and relationships that society offers its members, that combine to promote a sense of belonging (Clayton et al., 2013; Rowe, 2015). This framework directs attention to participation in society over the management of symptoms, challenging highly individualistic approaches which often underpin health and social care practice settings. An important feature of the citizenship framework involves the sharing of power between individuals and professionals (MacIntyre et al., 2019; Rowe, 2015; Rowe et al., 2001) and developing inclusive partnerships leading to user defined outcomes and knowledge (Beresford & Branfield, 2006; Miller, 2012).

Citizenship‐based programmes which have both individual and group‐based components have attempted to translate the 5Rs of citizenship into practice (Clayton et al., 2013; Rowe et al., 2007, 2012; Rowe & Ponce, 2020). Individuals with lived experience of a range of major life disrupting events (e.g. associated with psychiatric hospitalisation, offending or homelessness) who have engaged in citizenship programmes, have been found to experience an improved quality of life and increased sense of belonging within their communities as a result (Andersen et al., 2020; Clayton et al., 2013; Rowe et al., 2007). However, the extent to which the benefits experienced by individuals can be attributed to such interventions is difficult to capture using existing outcomes measures which are often focused on symptoms and illness‐based constructs (MacIntyre et al., 2019; Pelletier et al., 2017). This pioneering work led to the development of a U.S. citizenship measure and additional tools to guide citizenship‐enhancing practices (Bellamy et al., 2017; O’Connell et al., 2017; Rowe, 2015). Scotland offers its own political, social and cultural context that makes this work on citizenship particularly timely, with important lessons for and transferability to other socio‐cultural contexts. It can be argued that Scotland has a progressive policy environment that is receptive to ideas around citizenship and inclusion (Mooney et al., 2016). However, Scotland, like other parts of the United Kingdom, experiences significant inequalities with premature mortality rates more than four times higher in Scotland's most disadvantaged neighbourhoods (Scottish Government, 2020). This can be attributed in part to differential access to resources, healthcare, adequate housing and education as well as social and cultural opportunities (NHS Scotland, 2017), all of which were identified as being important aspects of citizenship in our earlier research (MacIntyre et al., 2021). Developing citizenship‐based practices is pertinent considering the significant changes to the way in which health and social care services are delivered in Scotland and other parts of Europe. There has been a renewed focus on joint working that has been mandated legislatively via the Public Bodies (Joint Working; Scotland) Act, 2014 and more recently a proposal to develop a National Care Service (Scottish Government, 2020) which will necessitate a shift in how we conceptualise and respond to the needs of those groups who have experienced significant periods of life disruption.

In this paper, we argue that a culturally and contextually specific measure of citizenship within the Scottish context is essential to establish the efficacy of citizenship‐based practices and to provide insight into the aspects of citizenship that could inform future research and citizenship‐based intervention development internationally (MacIntyre et al., 2021; Rowe et al., 2012). Measuring citizenship can help us to understand the ways in which people who have experienced major life disruptions have had their access to citizenship compromised or taken away (Cogan et al., 2021). By giving an indication of the areas of their live where things are going well (e.g., relationships) and the areas where an individual feels things are problematic (e.g., having a valued social role), we can begin to develop a greater understanding of the inclusion or marginalisation experienced by an individual.

We report on how we developed and tested the psychometric properties of a new measure of citizenship, the Strathclyde Citizenship Measure (SCM). The SCM is informed by our earlier work in which we developed an empirical model of citizenship (MacIntyre et al., 2019, 2021) adopting community participatory research methods, involving peer researchers with lived experience of significant life disruptions. Data collection involved focus groups with people who had experienced life disruptions and those who did not. This was followed by a process of concept mapping to identify patterns and relationships between the data. Using multidimensional scaling and hierarchical cluster analysis we identified five core domains of citizenship: ‘building relationships’, ‘autonomy and acceptance’, ‘access to services and supports’, ‘shared values and social roles’ and ‘civic rights and responsibilities’; representing the personal meanings of citizenship for participants, grounded in their own experiences and in their own words (MacIntyre et al., 2021). These domains map onto the 5Rs framework developed by Rowe (2015; rights, responsibilities, roles, resources and relationships as well as a sense of belonging) and highlight the interplay between the relational and structural aspects of citizenship whilst acknowledging the barriers that marginalised groups face in claiming their citizenship rights (Cogan et al., 2021; Rowe & Ponce, 2020). The development of the SCM will allow subsequent studies to accurately measure the effects and changes in citizenship experienced by people who engage in citizenship‐based programmes and/or research, as well as extending the applicability of citizenship across health and social care settings by providing a means for practitioners to explore lived experience of mental illness in a more holistic manner (O’Connell et al., 2017; Pelletier et al., 2017).

Below we outline the three phases of measure development in accordance with best practice (Boateng et al., 2018): (1) development of statement items, (2) item reduction and piloting, and (3) measure validation. We end with a discussion of the need for empirical work on the measurement of citizenship outside of the U.S., which aims to capture people's everyday interactions and experiences of citizenship (Hopkins & Blackwood, 2011); this is the gap our research has sought to address. Our colleagues (O’Connell et al., 2017; Rowe, 2015; Rowe et al., 2012) in the US have provided a foundation for this thinking with their ground‐breaking work on citizenship; this paper adds to this body of knowledge by undertaking empirical research on measuring citizenship within a Scottish context.

## 2 METHODS

We adopted a mixed method, community based participatory research approach (CBPR) involving peer researchers throughout the research process (see MacIntyre et al., 2018, 2019, 2021). Six peer researchers were recruited from a Service User and Carer Network in the University and via partner organisations in the third sector. Peer researchers were individuals who identified as having lived experience of major life disruptions (i.e. mental illness, justice experiences, substance misuse, homelessness) and were engaged throughout each of the stages of the research process (Cogan et al., 2021; MacIntyre et al., 2018, 2021). This included planning the study, collecting and analysing data, and reflecting on the research process. Evidence suggests that peer or service user involvement in the co‐production of research ensures that the research process is more sensitive to the needs of participants (Beresford, 2019; Davidson et al., 1999; O’Connell et al., 2017). It is a relevant, and important research framework that may guide the implementation of more effective, culturally appropriate, and socially just measure development (Collins et al., 2018). All quantitative data was managed and analysed using SPSS statistical software (version 23) and the qualitative data was managed using NVIVO (version 12). Data collection from phase 1 to 3 took place between August 2018 and January 2020.

### 2.1 Ethics

We received ethical approval for this research from the University Ethics Committee. All participants were recruited using convenience sampling using online platforms, Facebook, Twitter as well as recruitment posters and newspaper adverts in local community and third sector organisations. Informed consent was obtained prior to participants engaging with the research.

### 2.2 Phase 1: Development of statement items

A detailed description of our first phase of research—the development of statement items—is provided elsewhere (see MacIntyre et al., 2018, 2019, 2021) and briefly summarised below. While the initial focus of our work was on those who had experienced “life disruptions”, including those with mental ill health, resulting in delays in meeting culturally and socially defined life stages and social roles (Pickett et al., 1994), our intention was to develop a broad measure of citizenship that would have relevance to all persons, including those who did not identify as having experienced a life disruption. We recruited participants who were living with mental health problems, long‐term physical health problems, homelessness, addiction, recent experience of the justice system, and/or combinations of these experiences, as well as persons who had not experienced any such life disruptions. Our starting point was to ask participants ‘what does citizenship mean to you?’ using 10 focus groups (*n* = 77 participants). Each focus group was facilitated by peer researchers and rich discussions took place; the data was audio recorded and transcribed verbatim. This resulted in 708 preliminary statement items which were checked and rechecked for potential duplication of items, resulting in a total of 110 statement items. We used concept mapping techniques to identify and group participant‐generated statement items of citizenship (MacIntyre et al., 2019, 2021). Concept mapping involved multiple strategies to capture conceptual data on citizenship, integrating input from multiple stakeholders using multivariate data analyses to create a series of ‘maps’ or visual representations of the data (Davison, 1983; Everitt, 1980) that were used to guide the development of the SCM. Examples of statement items are ‘*Treating others as you would want to be treated*’ ‘*Having access to health services*,’ ‘*Giving back to my community*,’ and ‘*Challenging stigma and discrimination*’ (MacIntyre et al., 2021). Item analysis identified statements rated as most important and relevant to participants, resulting in 60 statements which were pilot tested in phase 2.

### 2.3 Phase 2: Item reduction and piloting

The second phase of developing the SCM involved piloting the statement items generated in phase 1 in order to further reduce the number of items and in doing so, ensuring that the questions were relevant, clear and measured what they were intended to measure (Boateng et al., 2018). This was achieved through assessing the 60‐item SCM and removing any items that did not contribute to the measure using stakeholder review and exploring the factor loadings of the statement items (Stanton et al., 2002). We piloted the SCM adopting a cross‐sectional design using an online, Qualtrics survey distributed via social media (Facebook, Twitter) and poster advertisements. Prior to consenting to take part, participants were provided with information about the study and the opportunity to ask questions through contacting the lead researchers. The participants were invited to anonymously complete the online version of the 60 item SCM in which they were asked to think about their lives at that time and rate statements such as ‘*You feel socially connected with others*’ and ‘*You take responsibility for looking after yourself*’ on a 5‐point Likert scale ranging from 1 (strongly disagree) to 5 (strongly agree). Upon completion of the SCM, participants were presented with a debrief and information on how to access local supports and services if needed. As recommended by Mundfrom et al. (2005), we sought to recruit between 3 and 20 participants per item—between 300 and 400 participants in total.

A sample of 407 participants completed the online survey. Inclusion criterion dictated that participants had to be aged 18 or over and to have lived in Scotland for 5 years or more. Seventy three percent of the participants were female (*n* = 297) and the age range was 18–88 years old (*M* = 43.72, *SD* = 15.24). In terms of ethnicity, the majority of participants were of white Scottish origin (*n* = 267; 65.6%), followed by those who identified as White British (*n* = 78, 19.2%) and White English (*n* = 10; 2.5%). In terms of life disruptions, participants reported to have experienced life disruptions associated with a diagnosed mental illness (*n* = 139), long term health condition (*n* = 69), addiction/substance misuse (*n* = 34), experience of the criminal justice system (*n* = 25), homelessness (*n* = 25), learning difficulties (*n* = 8) and seeking asylum (*n* = 1). Less than a quarter of the participants reported to have experienced no life disruptions (*n* = 89). The majority of the participants were in either full or part‐time paid work (*n* = 312) and identified as middle to traditional working class (see Table 1).

**TABLE 1:** Participant socio‐economic demographical characteristics for phase 2

Variables	*n* (407 total)
Age	*M* = 43.72 (*SD* = 15.24)
Gender	
Male	103 (25.3%)
Female	297 (73%)
Nonbinary	4 (1%)
Prefer not to say	3 (0.7%)
Ethnicity	
White Scottish	267 (65.6%)
White British	78 (19.2%)
White English	10 (2.5%)
White Irish	2 (0.5%)
Mixed Race	2 (0.5%)
Black African	1 (0.2%)
Pakistani	2 (0.5%)
Indian	1 (0.2%)
Bangladeshi	1 (0.2%)
Chinese	3 (0.7%)
Other	4 (1%)
Prefer not to say	1 (0.2%)
Life disruption	
Diagnosed mental illness	139 (34.2%)
Long term health issues	69 (17%)
Learning difficulties	8 (2%)
Homelessness	26 (6.4%)
Experience of the criminal justice system	25 (6.1%)
Addiction/substance abuse	34 (8.4%)
Seeking legal asylum	1 (0.2%)
Other	11 (2.7%)
Prefer not to say	9 (2.2%)
No life disruption	86 (46.1%)
Employment status	
Working full time	225 (55.3%)
Working part time	87 (21.4%)
Not currently in paid employment	28 (6.9%)
Voluntary work	33 (8.1%)
Student	59 (14.5%)
Carer	6 (1.5%)
Military veteran	2 (0.5%)
Other	41 (10.1%)
Prefer not to say	0 (0%)
Social status	
Elite	1 (0.2%)
Established middle class	77 (18.9%)
Technical middle class	70 (17.2%)
New affluent worker	29 (7.1%)
Traditional working class	117 (28.7%)
Service worker	17 (4.2%)
Poor	16 (3.9%)
Other	24 (5.9%)
Don't know	58 (14.3%)
Prefer not to say	11 (2.7%)

A principal components analysis (PCA) was used in order to consolidate items for which there was evidence of redundancy and to reduce the total number of items in the SCM. The PCA is a multistep process that involves evaluating the appropriateness of the dataset for analysis, extracting factors, rotating factors and interpreting the results (Tabachnick & Fidell, 2013). The adequacy of the dataset was determined by use of the Bartlett test for sphericity and the Kaiser–Meyer–Olkin (KMO) measure of sampling adequacy. Inspection of the correlation matrix showed that all variables had at least one correlation coefficient >0.3. The overall KMO measure was 0.94 with individual KMO measures all >0.9; these classifications are deemed ‘marvellous’ according to Kaiser (1974). Bartlett's test of sphericity was statistically significant (*p* < 0.001), indicating that the data was likely factorisable. The next step of the PCA was to extract factors and then perform an oblique rotation of these factors by use of oblimin rotation (delta = 0). The eigenvalue rule was used which states that factors that reach a value of greater than or equal to 1.0 will be retained for further evaluation (Carpenter, 2017). A scree plot test was used to determine the cut point in the curve and to evaluate the number of factors. PCA revealed twelve components that had eigenvalues greater than 1.0 and which explained 61.6% of the total variance, respectively. Visual inspection of the scree plot indicated that three components should be retained (Iantovics et al., 2018). In addition, a three‐component solution met the interpretability criterion. The research team used the pattern matrix to assess for overlap in the items in the SCM and the components that were extracted. Items with lower factor loading were removed. Subsequent item reduction analysis revealed that the removal of such items did not affect or improve the Cronbach's *α* (*α* = 0.95) of the SCM. Component loadings and communalities of the rotated solution are presented in Table 2.

**TABLE 2:** Component loadings and communalities of the rotated solution

	Factor 1	Factor 2	Factor 3
You try not to hurt others	0.749		
You look out for others	0.740		
You value the importance of 'giving back'	0.721		
You treat others fairly	0.714		
You help others when needed	0.709		
You share values with others	0.687		
You are respectful of others	0.685		
You recognise individuals who cannot contribute still have rights	0.653		
You do things to improve conditions for all	0.641		
You strive to ensure a better future for the next generation	0.594		
You speak up when you see someone being treated unfairly	0.590		
You live peacefully with others	0.579		0.372
You are a good neighbour	0.566		
You try to build relationships with others	0.557	0.440	
You are a law‐abiding citizen	0.557		
You care about others	0.551		
You understand your legal rights	0.527		
You take responsibility for the environment	0.526		
You believe living with others that are different from you is important	0.512		
You know what your entitlements are	0.492	0.330	
You believe it is important to pay your taxes	0.417		
You treat others are you want to be treated	0.413		
You are independent	0.395		
You have access to adequate housing	0.390		0.345
You feel like you belong		0.729	
You feel valued by others		0.718	0.342
You have a positive sense of identity		0.655	
You have a common purpose with others	0.368	0.648	
You feel included within your community		0.637	
You have social connections	0.379	0.604	
You get out and about in within your community		0.603	
You have a valued social role	0.328	0.574	
Others help you when needed		0.570	0.300
You have meaningful relationships		0.569	
You have opportunities to better yourself		0.560	
You have information about resources available to you	0.395	0.511	
You feel you are equal to others		0.500	0.391
You believe in yourself	0.306	0.499	
Others look out for you	0.360	0.496	0.310
You have a sense of hope for your future		0.496	0.328
You feel socially disconnected from others		−0.429	−0.346
You take responsibility for looking after yourself	0.328	0.353	
You are not discriminated against			0.787
Others discriminate against you			−0.708
Others are disrespectful of you			−0.701
You are not judged by others		0.325	0.642
Others treat you equally			0.637
Others treat you fairly		0.313	0.628
You feel stigmatised by others			−0.622
You feel accepted by others		0.545	0.576
Others are accepting of you		0.407	0.568
You have privacy in your life			0.567
You feel safe in your community			0.521
You have adequate health services			0.422
You have a ‘safety net’ during hard times		0.393	0.409
You have access to adequate education		0.311	0.395
You feel confident that if you leave the country you will be able to return	0.314		0.390
You are represented politically			0.348
You have access to adequate transport			0.309
You believe it is important for people in your country to speak the same language			—

**FIGURE 1:**
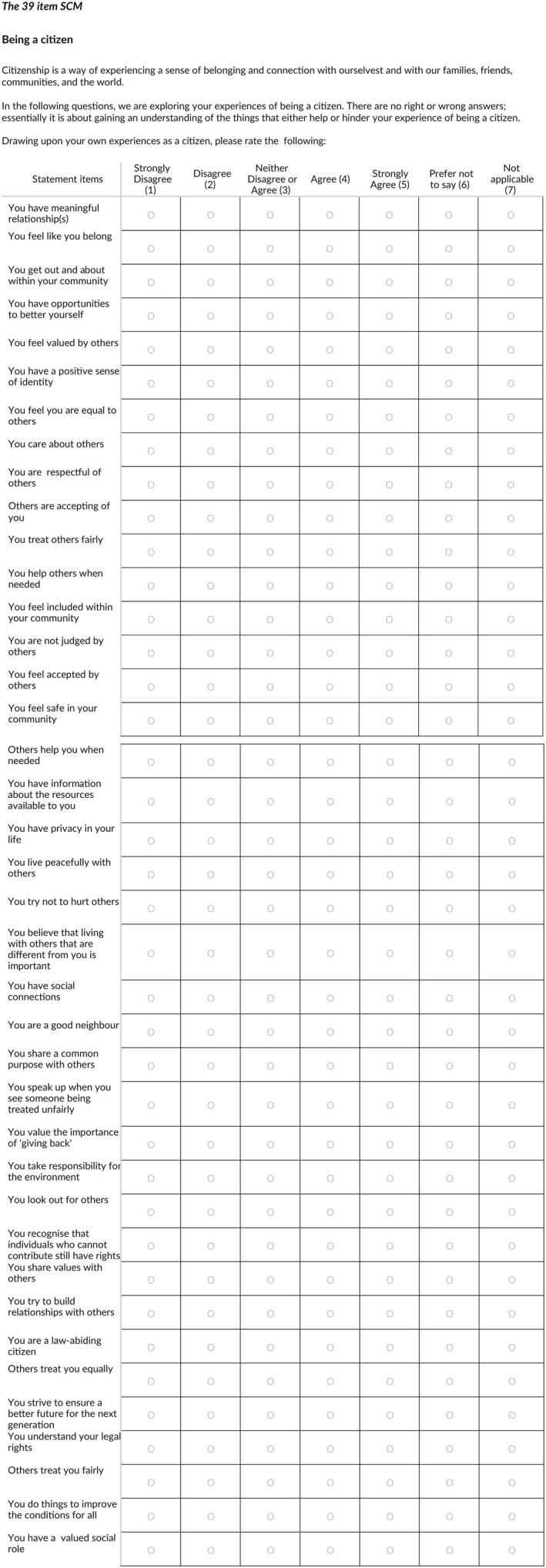
The 39 item Strathclyde Citizenship Measure (SCM)

A new, shorter, 39‐item version of the SCM was created (see Figure 1).

### 2.4 Phase 3: Measure validation

The third phase involved administering the 39‐item version of the SCM to a sample of participants using the same inclusion criteria as in phase 2. The aim was to assess the reliability, concurrent validity, convergent validity, discriminant validity and dimensionality of the SCM. We sought to recruit 3–20 participants per item, aiming for a sample of 200–300 participants (Mundfrom et al., 2005). Participants were invited to complete the 39 item SCM in which participants are asked to think about their lives right now and rate statements like ‘*You feel like you belong*’ and ‘Y*ou are not judged by others*’ on a 5‐point Likert scale ranging from 1 (strongly disagree) to 5 (strongly agree). In addition to the 39‐item SCM, participants were invited to complete a series of psychometrically valid measures that, we hypothesised were moderately and positively associated with scores on the SCM.

#### 2.4.1 The Warwick‐Edinburgh Mental Well‐Being Scale (Tennant et al., 2007)

The Warwick‐Edinburgh Mental Well‐Being Scale (WEMWBS) is a scale of 14 positively worded items for assessing mental wellbeing using a likert‐type response format (1 = none of the time to 5 = all of the time). Questions include items such as ‘*I’ve been feeling optimistic about the future*’. Higher scores on the WEMWBS indicate higher levels of wellbeing. WEMWBS has internal consistency reliability of 0.70 and Cronbach's α of 0.91. Test–retest reliability after 1 week in a student population was 0.83, indicating high reliability for scale (Tennant et al., 2007).

#### 2.4.2 The Depression, Anxiety and Stress Scale (Lovibond & Lovibond, 1995)

The Depression, Anxiety and Stress Scale (DASS‐21) is a set of three self‐report sub‐scales designed to measure the emotional states of depression, anxiety, and stress. Each of the three DASS‐21 scales contains 7 items, divided into subscales with similar content. Questions include items such as ‘*I found it hard to wind down*’. Higher scores on each of the sub‐scales indicates higher levels of depression, anxiety and stress. DASS21 has internal reliability of 0.94 and Cronbach's α ranged between 0.86 and 0.90 (Gloster et al., 2008).

#### 2.4.3 The Sense of Belonging Instrument (Hagerty & Patusky, 1995)

The Sense of Belonging Instrument (SOBI‐A) scale represents a single factor of the precursors of sense of belonging, which relates to the motivation and capability for developing a sense of belonging. The SOBI‐A (Hagerty & Patusky, 1995) consists of 9 items scored on a four‐point Likert‐type scale (4 = strongly agree to 1 = strongly disagree). Questions include items such as ‘*I want to be part of things going on around me*’. Reliability was evaluated and the internal consistency reliability for each group was 0.72, 0.63 and 0.76 and Cronbach's *α* was 0.72 (Hagerty et al., 1996). Stability was examined through test–retest reliability with the student group which was 0.66 for over an 8‐week period.

#### 2.4.4 The Big Five Inventory (Rammstedt & John, 2007)

The Big Five Inventory (BFI‐10) looks to measure personality traits. The measure includes five factors made of 10 items; each factor entails two items: extraversion (items 1 and 6), agreeableness (items 2 and 7), conscientiousness (items 3 and 8), neuroticism (items 4 and 9) and openness (items 5 and 10). The scale for the assessment of the items is from 1—strongly disagree to 5—strongly agree, and five of the items have a reversed score. Questions include statements such as ‘*I see myself as someone who is reserved*’. Reliability was checked by means of a second test after a period of 6–8 weeks. The test–retest correlations varied between 0.68 and 0.84 suggesting that the BFI‐10 has acceptable retest reliability and The Cronbach *α* coefficients obtained varied between 0.03 and 0.75 (Balgiu, 2018).

#### 2.4.5 The Personal Social Capital Scale 16 (Archuleta & Miller, 2011)

The Personal Social Capital Scale (PSCS) is based on 4 components including: size of a person's network connections, trustworthiness of their network members, resource‐ownership of the network members, and reciprocity with network members (Archuleta & Miller, 2011). Questions include items such as ‘*How do you rate the number of cultural, recreational and leisure groups/organisation in your community?*’ The psychometric properties of the PSCS indicated acceptable overall (α=0.85) and subscale (bonding *α* = 0.83; bridging *α* = 0.85) reliability. The scale demonstrates convergent validity with a psychological sense of community (*r* = 0.44; Archuleta & Miller, 2011).

As noted above, we hypothesised that citizenship scores would be positively related to each of these measures. Based on previous work (O’Connell et al., 2017), however, we expected that scores on citizenship would be more positively correlated with sense of belonging, social capital and wellbeing scores than with personality constructs. Finally, participants were presented with a debrief on the purpose of the survey and information on support services if needed.

A sample of 280 participants completed the anonymous online survey: 30.7% (*n* = 86) were male, 68.2% (*n* = 191) were female, 1.1% (*n* = 3) were non‐binary. The age of participants ranged from 18–85 (*M* = 42.78, *SD* = 16.57). The ethnicity, employment status and social class of participants was reported and can be viewed in Table 3.

**TABLE 3:** Participant socio‐economic demographical characteristics for phase 3

Variables	*n* (280 total)
Age	*M* = 42.78 (*SD* = 16.57)
Gender	
Male	86 (30.7%)
Female	191 (68.2%)
Nonbinary	3 (1.1%)
Prefer not to say	0 (0%)
Ethnicity	
White Scottish	177 (63.2%)
White British	68 (24.3%)
White English	5 (1.8%)
White Irish	2 (0.7%)
Afro Caribbean	6 (2.1%)
Black African	2 (0.7%)
Pakistani	3 (1.1%)
Indian	0 (0%)
Bangladeshi	0 (0%)
Chinese	1 (0.4%)
Other	2 (0.7%)
Prefer not to say	1 (0.2%)
Life disruption	
Diagnosed mental illness	57 (20.4%)
Long term health issues	43 (15.4%)
Learning difficulties	9 (3.2%)
Homelessness	1 (0.4%)
Experience of the criminal justice system	4 (1.4%)
Addiction/substance abuse	11 (3.9%)
Seeking legal asylum	0 (0%)
Other	4 (1.4%)
Prefer not to say	2 (0.7%)
No life disruption	174 (62.1%)
Employment status	
Working full time	131 (46.8%)
Working part time	54 (19.3%)
Not currently in paid employment	16 (5.7%)
Voluntary work	8 (2.9%)
Student	44 (15.7%)
Carer	0 (0%)
Military veteran	2 (0.7%)
Other	27 (9.6%)
Prefer not to say	0 (0%)
Social status	
Elite	3 (1.1%)
Established middle class	72 (25.7%)
Technical middle class	59 (21.1%)
New affluent worker	21 (7.5%)
Traditional working class	73 (26.1%)
Service worker	8 (2.9%)
Poor	6 (3.1%)
Other	14 (5%)
Don't know	22 (7.9%)
Prefer not to say	2 (0.7%)

## 3 FINDINGS

### 3.1 Reliability

We assessed reliability in order to ascertain if there was internal consistency within the SCM using Cronbach's *α*. A high level of reliability was found (*α* = 0.94).

### 3.2 Concurrent, convergent and discriminant validity

We assessed the concurrent validity by correlating Citizenship (SCM) with Sense of Belonging (SOBI). The SCM mean score correlated moderately strongly with the corresponding mean in the SOBI, indicating good concurrent validity. Correlation analysis revealed that overall mean scores on the SCM were positively associated with scores on Mental Health (DASS21), Wellbeing (WEMWBS) and Social Capital (PSCS‐16) suggesting good convergent validity (see Table 4).

**TABLE 4:** Correlations for concurrent, convergent and discriminatory validity

		1.	2.	3.	4.	5.	6.
1. Citizenship	Pearson correlation	1	0.457^a^	−0.398^a^	0.140^b^	0.530^a^	0.155^a^
	Sig. (two‐tailed)		0.000	0.000	0.019	0.000	0.009
	*N*	282	280	280	280	280	280
2. Sense of belonging	Pearson correlation	0.457^a^	1	−0.037	0.036	0.323^a^	0.308^a^
	Sig. (two‐tailed)	0.000		0.536	0.545	0.000	0.000
	*N*	280	280	280	280	280	280
3. Mental health	Pearson correlation	−0.398^a^	−0.037	1	−0.068	−0.436^a^	−0.078
	Sig. (two‐tailed)	0.000	0.536		0.257	0.000	0.195
	*N*	280	280	280	280	280	280
4. Wellbeing	Pearson correlation	0.140^b^	0.036	−0.068	1	0.079	0.120^b^
	Sig. (two‐tailed)	0.019	0.545	0.257		0.185	0.044
	*N*	280	280	280	280	280	280
5. Social capital	Pearson Correlation	0.530^a^	0.323^a^	−0.436^a^	0.079	1	0.109
	Sig. (2‐tailed)	0.000	0.000	0.000	0.185		0.068
	*N*	280	280	280	280	280	280
6. Personality	Pearson correlation	0.155^a^	0.308^a^	−0.078	0.120^b^	0.109	1
	Sig. (two‐tailed)	0.009	0.000	0.195	0.044	0.068	
	*N*	280	280	280	280	280	280

^a^ Correlation is significant at the 0.01 level (two tailed).

^b^ Correlation is significant at the 0.05 level (two tailed).

Discriminant validity was assessed by examining the correlations between the mean SCM total score with personality (BFI‐10). As expected, these correlations were found to be weaker than those observed with the SOBI, WEMWBS, DASS21 and PSCS‐16 indicating sound discriminant validity.

### 3.3 Dimensionality of the SCM

An exploratory factor analysis was used to determine the dimensionality of the SCM (Barendse et al., 2015). Inspection of the correlation matrix showed that all variables had at least one correlation coefficient >0.3. The overall KMO measure was 0.906 with individual KMO measures all >0.9, classifications of ‘marvellous’ according to Kaiser (1974). Bartlett's test of sphericity was statistically significant (*p* < 0.001). Visual inspection of the scree plot indicated that three components should be retained (Cattell, 1966), explaining 37.7% of the total variance. A Varimax orthogonal rotation was employed to aid interpretability. The rotated solution exhibited 'simple structure' (Wherry & Thurstone, 1948). The interpretation of the data was consistent with the attributes the SCM was designed to measure with strong loadings of ‘*Perceptions of the quality of relationships with others*’ (How relationships with others are experienced and understood. For example, feeling accepted, valued, included) on Component 1, ‘*Actions taken to build relationships with others*’ (Actions taken to build, sustain and strengthen relationships with others. For example, caring and/or helping for others) on Component 2, and ‘*Responsibility towards others*’ (Altruistic behaviours, accountability or actions to others. For example, treating others respectfully and seeking to cause no harm) on Component 3. Component loadings and communalities of the rotated solution are presented in Table 5.

**TABLE 5:** Component loadings and communalities of the rotated solution

Questions	Perceptions of relationships with others	Actions relating to relationships with others	Responsibilities to others
You feel accepted by others	0.807		
You feel valued by others	0.733		
Others are accepting of you	0.724		
You feel included within your community	0.711		
Others treat you equally	0.699		
You feel like you belong	0.692		
Others treat you fairly	0.646		
Others help you when needed	0.643		
You have social connections	0.642		
You are not judged by others	0.622		
You feel you are equal to others	0.604		
You feel safe in your community	0.603		
You have a positive sense of identity	0.578		0.320
You get out and about in your community	0.554		
You have a valued social role	0.539	0.381	
You live peacefully with others	0.529	0.347	
You have meaningful relationships	0.527		0.340
You have opportunities to better yourself	0.525		
You have privacy in your life	0.444		
You have information about the resources available to you	0.431	0.309	
You do things to improve conditions for all		0.727	
You strive to ensure a better future for the next generation		0.669	
You value the importance of giving back		0.599	
You look out for others		0.581	0.351
You try to build relationships with others	0.395	0.548	
You believe living with others that are different to you is important		0.542	
You share values with others		0.523	
You speak up when you see someone being treated unfairly		0.519	
You share a common purpose with others	0.444	0.516	
You are a good neighbour	0.353	0.457	
You take responsibility for the environment		0.457	
You understand your legal rights	0.323	0.371	
You are respectful of others			0.749
You treat others fairly			0.725
You care about others			0.692
You help others when needed		0.310	0.608
You try not to hurt others		0.369	0.577
You are a law‐abiding citizen		0.303	0.398

Note

*Perceptions of the quality of relationships with others’* (How relationships with others are experienced and understood. For example, feeling accepted, valued, included). ‘*Actions taken to build relationships with others*’ (Actions taken to build, sustain and strengthen relationships with others. For example, caring and/or helping for others). ‘*Responsibility towards others*’ (Altruistic behaviour, accountability or actions to others. For example, treating others respectfully and seeking to cause no harm).

## 4 DISCUSSION

The study reports that the SCM is a psychometrically sound measure that captures the multiple dimensions of citizenship that people experience but that, until now, have been difficult to operationalise and measure within the Scottish context. This paper builds upon previous work exploring the development of a measurement of citizenship within the US (O’Connell et al., 2017; Rowe et al., 2012) and provides a novel contribution to the growing body of work that aims to operationalise and measure citizenship across health and social care settings (Bromage et al., 2021; Nesse, Gonzalez, et al., 2021). The CBPR and concept mapping procedures used to create the statement items in phase 1 of developing the SCM support the relevance of items included in the SCM to individuals who have experienced various life disrupting experiences and differing degrees of marginalisation, as well as for those who do not primarily identify as having experienced such a disruption (Cogan et al., 2021; MacIntyre et al., 2019; O’Connell et al., 2017). Through this process, we have gained a more nuanced understanding of citizenship which is grounded in the lived experiences of participants, that is related to, but distinct from mental health, well‐being, sense of belonging and social capital. In testing the psychometric properties of the SCM, it was shown to have good internal consistency and good levels of convergent, concurrent and discriminant validity; the SCM is therefore a sound psychometric measure. This paper highlights the importance of the relational aspects of citizenship that our participants articulated in our earlier work (MacIntyre et al., 2021) and as captured in the component loadings and communalities of the rotated solution. Indeed, the current study provides convincing evidence for three core elements of citizenship; ‘perceptions of the quality of relationships with others’; ‘actions taken to build relationships with others’ and ‘responsibility towards others’ that can be mapped onto our model of citizenship discussed earlier (MacIntyre et al., 2018, 2021). The three core elements highlighted here suggest that how one views one's relationships with others and the extent to which one feels agency to take actions to promote and invest in their relationships are critical factors that may predict one's sense of citizenship. This is in line with the model of citizenship that we developed whereby positive relationships with others was emphasised strongly by all participants, regardless of whether they had experienced a life disruption. It also captures the action‐oriented nature of citizenship encapsulated by participants’ discussions of the importance of carrying out specific tasks or adopting specific roles to achieve recognition, value and respect from others. This fits well with the notion of ‘giving back’, evident in earlier citizenship studies in the US (Bellamy et al., 2017; Rowe, 2015) and more recent work on collective citizenship by Quinn et al. (2020). This suggests that citizenship can play an important role in reducing isolation and promoting social capital (Clayton et al., 2013). Indeed, those who feel valued are more likely to make contributions and give back, whereas making contributions increases opportunities for feeling valued (Prilleltensky & Prilleltensky, 2021).

Social capital theory discusses how individuals can enhance their participation in society through bonds, bridges and links (Putman, 2000). It is clear from this study that a focus on citizenship can help people experiencing life disruptions enhance and build their relationships. The findings from this study highlight the importance of relationships and a sense of belonging for promoting citizenship thus adding important empirical support to the earlier work of Rowe (2015) and confirming the findings from our earlier work (Cogan et al., 2021; MacIntyre et al., 2019, 2021). This finding also supports the work of Hagerty et al. (1992) who has argued that sense of belonging is a ‘vital mental health concept’ and Hill (2006) who suggests that a sense of belonging and connectedness is particularly important for groups who have previously been marginalised. Indeed, Hill (2006) has argued that a sense of belonging can promote inter‐personal relationships as well as individual, family and community well‐being. Importantly Torgerson et al. (2018) suggest that a sense of belonging offers an important ‘buffering’ effect against adversity and childhood trauma suggesting that relationship‐based and trauma‐informed practices are vital.

We argue that adopting relationship‐based practices that focus on the individual and those around them in their broader social context can play a significant role in supporting some of the most marginalised people in our communities to develop their sense of citizenship and in turn their sense of belonging, which corresponds to earlier work on the value of citizenship (Clayton et al., 2013; Erdal et al., 2018). This will require a cultural shift that will involve professionals moving beyond a traditional focus on individual problems or deficits. There has been some resistance to this as has been elaborated on elsewhere (see e.g., Clayton et al., 2020; Eiroa‐Orosa & Rowe, 2017; Ponce et al., 2016) with some practitioners expressing concerns about resource implications, remit, and boundaries. The current political and social climate in Scotland, with a renewed focus on partnership working and collaboration would appear ripe for such a change. As highlighted earlier in this paper, Scotland's progressive policy environment focused on promoting inclusion (Mooney et al., 2016) is well suited to incorporating the concept of citizenship into its policy agenda across a range of different areas of policy. This also fits well with the Scottish Government's stated policy commitment to tackling health inequalities (Walsh et al., 2020).

Currently, we are conducting further research to understand more about the latent factors underlying the conceptually derived dimensions of the SCM via confirmatory factor analysis (CFA) and once understood, will explore the possibility of creating a briefer measure. We are also collaborating with colleagues in the third sector, who are engaged in citizenship‐based practices, to test the feasibility of using the SCM in intervention planning and goal setting with service users. Ongoing work in the US to translate the Citizenship Measure into a tool (Bellamy et al., 2017) has provided useful data on the utility of such a tool from a practitioner perspective. It was reported that any such tool must be manageable in terms of length and that training on how to use the tool is essential. Practitioners suggested that the citizenship tool aided reflective practice and promoted open dialogue between staff and people who use services although they felt they needed more guidance on how to support people after using it. We are building upon this work through collaborating with a Citizenship Measure Working Group within a third sector organisation that are piloting the SCM with service users who have experienced a range of major life disruptions as well as mental health practitioners. The aim of this working group is to help establish guidelines and best practice for using the SCM within practice and community settings and as a tool to facilitate open dialogues concerning the multi‐dimensions of citizenship.

Future work will explore the psychometric properties and feasibility of the SCM in more diverse populations (e.g., people from BAME groups, children and young people, people with learning disabilities) and across a range of socio‐cultural contexts (e.g. low‐ and middle‐income countries). Longitudinal research is additionally required to establish the ability of the SCM to predict objective measurements of engagement in citizenship‐based activities such as volunteering, and community‐based activities. The SCM can be used to evaluate the long‐term outcomes of intervention programmes that aim to increase a sense of citizenship across health and social care practice and community settings.

This work may also provide us with a greater understanding of the individual recovery process by identifying the aspects of citizenship that might contribute to an individual's life in the community that the citizenship framework aims to support, thus enhancing individual recovery (Davidson et al., 2021; Rowe & Davidson, 2016). This would act as a key marker in our efforts to realise this vision of citizenship as an individual and collective concept in practice given the development of psychometrically valid measures of citizenship at the individual level alongside our efforts to develop and test citizenship‐based practices at the community level (Cogan et al., 2021; O’Connell et al., 2017; Quinn et al., 2020). Practical findings stemming from such work will inform intervention development, research, and training for health and social care workers aiming to engage in citizenship‐based practices in partnership with service users and carers. This may result in more meaningful service user and carer involvement in the co‐production of health and social care services, policy and research (Beresford, 2019). Further, as these findings are correlational, they do not provide an indicator of directionality of effect. Future research is needed to determine whether higher scores on the SCM predict more positive mental health, well‐being, sense of belonging, and social capital or vice versa. A more nuanced and contextual understanding of citizenship could further be developed through exploring whether people feel a sense of increased citizenship in one context but neglected in another. For instance, recent research developing a multidimensional tool of feeling valued and adding value, the Mattering in Domains of Life Scale (Scarpa et al., 2021), reported that mattering may express itself differently in various communities. Research exploring citizenship and mattering across diverse domains of life may help us understand these important social and contextual differences. Finally, future studies should examine the relationship between the SCM and other measures of related constructs, such as recovery, social capital, occupational meaningfulness, belonging, feeling valued and sense of mattering across a range of socio‐cultural contexts (Hamer et al., 2019; Harper et al., 2017; Pelletier et al., 2020). We believe that the SCM, whilst developed within the Scottish context, has wider applicability. We recommend that researchers or practitioners who want to use it, or develop it further, be mindful of cultural and linguistic nuances and diversities. Having a better understanding of these important differences will help us understand and develop best practices in citizenship‐based care, policies and research.

### 4.1 Limitations

This study has several limitations. First, since participants self‐selected through convenience sampling rather than being randomly selected, they may have had stronger feelings about citizenship than those in the larger population in general. Second, while the statement items for the SCM were developed across multiple groups of people, reliability and validity were assessed using data from an anonymous online Qualtrics survey, self‐reporting on their socio‐demographic characteristics without objective verification. Third, participants who identified as experiencing major life disruptions were so classified only in relation to specified life disrupting events, thus suggesting the need for further validation studies with a wider range of life disruptions in this regard.

## 5 CONCLUSIONS

The research described in this paper, and other research currently underway, establishes a platform for future work to support and enhance the citizenship of people who have experienced marginalisation. The SCM is a new and novel measure with sound psychometric properties including excellent reliability as well as good convergent, discriminant and concurrent validity. This, therefore, invites consideration of the environments in which the SCM can be effectively utilised across health and social care settings. In addition, the SCM displays a 3‐factor model indicating that the ‘relationships’ dimension of citizenship appears to be a strong influencing factor on citizenship within a Scottish context. This fits well with notions of social capital and promoting social inclusion across a range of policy areas within a progressive policy environment. However, future work should undertake a CFA to fully establish how well this model fits this factor structure and to compare it across cultures and contexts. We envisage that the SCM will enable policy‐makers, practitioners and/or people who are users of services to meaningfully discuss and capture the aspects of citizenship that are important to them, allowing them to record changes experienced over time. Those who have experienced major life disruption(s) face multi‐level barriers to citizenship (Cogan et al., 2021). The SCM by helping to raise awareness of such barriers and providing a means of measuring citizenship, can assist individuals, practitioners and policymakers in taking steps to overcome these barriers, thus promoting citizenship as practice. Such an approach is likely to have significant implications for mental health research, policy and practice.

## CONFLICT OF INTEREST

There are no known conflicts of interest for any of the authors.

## AUTHOR CONTRIBUTIONS

Dr Nicola Cogan (corresponding author): Conceptualisation, methodology, formal analysis, investigation, writing original draft, writing reviewing and editing, visualisation, supervision, project administration. Dr Gillian MacIntyre: Conceptualisation, methodology, formal analysis, investigation, writing reviewing and editing, visualisation, supervision, project administration, funding acquisition. Dr Ailsa Stewart: Conceptualisation, methodology, formal analysis, investigation, reviewing and editing, visualisation, project administration, funding acquisition. Hilary Harrison‐Millan: Methodology, formal analysis, investigation, writing reviewing and editing. Karen Black: Reviewing and editing. Neil Quinn: Reviewing and editing, funding acquisition. Professor Michael Rowe: Conceptualisation, methodology, reviewing and editing. Dr Maria O’ Connell: Conceptualisation, formal analysis, methodology, reviewing and editing.

## Data Availability

Anonymised data available on request due to privacy/ethical restrictions.
